# PPP1R12B inhibits cell proliferation by inducing G0/G1 phase arrest via PAK2/β-catenin axis in hepatocellular carcinoma

**DOI:** 10.3389/fcell.2025.1621705

**Published:** 2025-06-19

**Authors:** Yangqianwen Zhang, Shuowu Liu, Mixue Bai, Zihan Zhao, Shan Wang, Meiyu Bao, Jinxia Bao, Siyun Shen, Shuang Lu, Ying Xiong, Gaoxiang Gu, Hongyang Wang, Lei Chen

**Affiliations:** ^1^ The International Cooperation Laboratory on Signal Transduction, National Center for Liver Cancer, Eastern Hepatobiliary Surgery Hospital, Naval Medical University, Shanghai, China; ^2^ Model Animal Research Center, Medical School, Nanjing University, Nanjing, China; ^3^ Longhua Hospital, Shanghai University of Traditional Chinese Medicine, Shanghai, China

**Keywords:** PPP1R12B, PAK2/β-catenin axis, hepatocellular carcinoma, cell proliferation, cell cycle

## Abstract

Protein phosphatase 1 regulatory subunit 12B (PPP1R12B) is a regulatory subunit of protein phosphatase 1. While our previous study identified the inhibitory role of PPP1R12B in hepatocellular carcinoma (HCC), the precise molecular mechanisms underlying its anti-proliferative effects remain unclear. Herein, we demonstrated that PPP1R12B expression is significantly downregulated in HCC tissues and serves as an independent prognostic marker for favorable patient outcomes. Additionally, overexpression and silence of PPP1R12B experiments showed that PPP1R12B overexpression restricted cell proliferation and colony formation *in vitro*, and inhibited xenografted tumor growth *in vivo*, while its knockdown had opposite effects. Mechanistically, PPP1R12B could interact with p21-activated kinase 2 (PAK2) to suppress β-catenin expression and phosphorylation at Ser675, thereby impeding its nuclear translocation and subsequent transcriptional activation of Cyclin D1. This cascade culminated in G0/G1 phase cell cycle arrest. Furthermore, analysis of TCGA-HCC datasets confirmed inverse correlations between PPP1R12B and PAK2 or CTNNB1 (β-catenin) expression. Collectively, our findings elucidated a novel tumor-suppressive role of PPP1R12B in HCC through modulation of the PAK2/β-catenin/Cyclin D1 axis.

## 1 Introduction

Primary liver cancer (PLC) ranks as the third leading cause of cancer-related deaths and the sixth most commonly diagnosed cancer worldwide (Bray et al., 2024). Hepatocellular carcinoma (HCC) constitutes 75%–85% of PLC cases (Rumgay et al., 2022), with over half of global HCC diagnoses occurring in China (Hepatocellular carcinoma, 2021). Despite advances in therapeutic strategies, HCC remains a high mortality, with projected annual deaths exceeding one million by 2030 globally (Pinter et al., 2023). The limited efficacy of current treatments underscores the urgent need to identify novel molecular targets for HCC intervention. Our previous study identified protein phosphatase 1 regulatory subunit 12B (PPP1R12B) 3′UTR mutation as one of the earliest mutational events during HCC evolution (Chen et al., 2024), underscoring its significance.

PPP1R12B, also designated as myosin phosphatase target subunit 2, functions as a regulatory component of protein phosphatase 1 (PP1). While its role in cardiac physiology has been extensively characterized (Mizutani et al., 2010; Chang et al., 2015; Hitsumoto et al., 2023; Hu et al., 2023), emerging evidence suggests context-dependent functions in oncogenesis. Notably, PPP1R12B acted as a tumor suppressor in colorectal cancer (Zhou et al., 2008; Ding et al., 2019; Tan et al., 2022) and esophageal cancer (Chen et al., 2022), but as an oncogenic factor in Wilms’ tumor (He et al., 2021) and breast cancer (Fokkelman et al., 2016). Our prior work confirmed that PPP1R12B suppressed proliferation, migration, invasion, and self-renewal of HCC cells (Chen et al., 2024), yet the precise regulatory mechanisms remained unknown.

The p21-activated kinase (PAK) family comprises conserved serine/threonine kinases (Manser et al., 1994). PAK2, a member of PAKs, could phosphorylate myosin light-chain kinase, mitogen-activated protein kinase-interacting kinase, and Myc protein, regulating cell proliferation, apoptosis, and cytoskeletal dynamics (Goeckeler et al., 2000; Huang et al., 2004; Orton et al., 2004). Additionally, PAK2 modulated endothelial cell migration, proliferation, and angiogenesis (Han et al., 2024), played a role in pancreatic exocrine secretion (Ramos-Alvarez and Jensen, 2025), and was implicated in multiple cancer-related signaling pathways (Chen et al., 2025). Elevated PAK2 expression correlated with advanced tumor progression, poor prognosis, and clinical staging in malignancies (Cho et al., 2012; Gao et al., 2014; Shuang et al., 2024).

β-catenin is a central transducer of the canonical Wnt/β-catenin signaling pathway. Upon activation, β-catenin would translocate to the nucleus, bind to T-cell factor/lymphoid enhancer-binding factor (TCF/LEF), and induce transcription of downstream oncogenic genes (e.g., *CCND1, c-MYC*) (Xu et al., 2022). Genomic studies revealed aberrant Wnt/β-catenin activation in approximately one-third of tumors (Giles et al., 2003). In HCC, mutations in CTNNB1 (encoding β-catenin) are recognized as key genetic events (Torrecilla et al., 2017). Wnt/β-catenin activation was closely linked to HCC stemness, progression, metastasis, and drug resistance (Yamashita et al., 2007; Liu et al., 2010; Liu Y. et al., 2015; Khalaf et al., 2018; Liao et al., 2020).

In this study, we integrated clinical data with functional experiments to delineate the molecular basis of PPP1R12B-mediated tumor suppression in HCC proliferation. We demonstrated that PPP1R12B inhibited HCC cell proliferation by physically interacting with PAK2 to suppress expression and Ser675 phosphorylation of β-catenin. This blocked β-catenin nuclear translocation and decreased Cyclin D1 expression, which induced cell cycle G0/G1 phase arrest. Moreover, we found that PPP1R12B predicted a favorable prognosis in HCC patients, and a negative correlation between PPP1R12B and PAK2 or CTNNB1 expression was observed in TCGA-HCC patients. These findings not only expand our understanding of HCC pathogenesis but also identify potential therapeutic targets for clinical intervention.

## 2 Materials and methods

### 2.1 Cell culture and maintenance

Human liver cancer cell lines (PLC/PRF/5, CSQT-2, HepG2, Huh7, MHCC-97H) along with the normal liver cell line HHL5 were acquired from the Shanghai Cell Bank, Chinese Academy of Sciences. All cell lines were maintained in Dulbecco’s Modified Eagle Medium (DMEM; Basal Media) supplemented with 10% fetal bovine serum (FBS; Gibco) and antibiotic-antimycotic solution (NCM Biotech). Cultures were incubated at 37°C in a humidified atmosphere containing 5% CO_2_.

### 2.2 Clinical specimen collection

HCC specimens and matched adjacent non-tumor tissues were obtained from patients undergoing resection at Eastern Hepatobiliary Surgery Hospital. The study protocol received ethical approval from the hospital’s Institutional Review Board (protocol code EHBHKY2018-1-001 and approved on the 6 June 2018). Written informed consent was obtained from all participants prior to sample collection. All specimens were anonymized and processed according to standard ethical guidelines.

### 2.3 Cell proliferation assessment

Cell viability was quantified using the Cell Counting Kit-8 (CCK-8; Absin) following manufacturer specifications. Briefly, cells were seeded in 96-well plates (1–1.5 × 10^3^ cells/well) and cultured for indicated durations. Following cell adhesion, CCK-8 reagent (10 μL/well) was added and incubated for 1 h at 37°C before measuring absorbance at 450 nm using a Synergy H1 microplate reader (BioTek) to obtain the Day 0 value. Repeat the assay at 24 h, 48 h, 72 h, 96 h, and 120 h to collect data for Day1 through Day 5. Data were normalized to baseline measurements (Day 0) and expressed as fold-change relative to controls.

### 2.4 Colony formation assay

For colony formation analysis, 1.5 × 10^3^ cells were plated in 6-well plates and cultured for 7–14 days with medium replacement every 72 h. Colonies were fixed with 4% paraformaldehyde (15 min), stained with 0.1% crystal violet (15 min), and quantified the stained colonies by measuring the clone area using image analysis software. Results represent fold-change compared to control groups.

### 2.5 Genetic manipulation of cell lines

The lentiviruses of short hairpin RNA (shRNA) targeting PPP1R12B and scramble shRNA (ShCtrl) were obtained (Genechem Co.), and transfected into PLC/PRF/5, CSQT-2 and HHL5 cell lines according to the manufacturer’s instructions. ShRNA sequences were as follows: ShPPP1R12B:5′-CcggAAGGATCTTCTTCTGGAGCAACTCGAGTTGCTCCAGAAGAAGATCCTTTTTTTg-3′; ShCtrl: 5′-CcggTTCTCCGAACGTGTCACGTCTCGAGACGTGACACGTTCGGAGAATTTTTg-3′. Overexpression lentiviral particles of PPP1R12B (NCBI Reference Sequence: NM_002481) and control lentiviral particles were constructed (Genechem Co.) and transfected into Huh7, HepG2 and MHCC-97H cell lines according to the manufacturer’s instructions. For overexpression studies, the full-length PPP1R12B coding sequence was amplified using primers (Sense: 5′-CCAACTTTGTGCCAACCGGTCGCCACCATGGCGGAACTGGAGCACCTAGGAGGG-3′; Antisense: 5′-GTCAATGCCAACTGAGCTTCTTGGACAGTTTGCTGATGAC-3′) and cloned into the GV341 lentiviral vector (AgeI/NheI sites). Since the lentiviral vector carries a puromycin resistance gene, transfected cells were selected with puromycin (2–5 μg/mL, concentration optimized for each cell line) for 3–10 days. Protein lysates of transfected cells were subsequently collected and subjected to Western blot analysis to confirm PPP1R12B overexpression/knockdown efficiency. Puromycin selection pressure was applied intermittently (every other passage) to maintain transduction efficiency (maintenance at half the working concentration).

### 2.6 RNA interference

PAK2 knockdown was achieved using siRNA duplexes (Sense: 5′-CCGGAUCAUACGAAAAUCAATT-3'; Antisense: 5′-UUGAUUUCGUAUGAUCCGGTT-3′) transfected with Lipofectamine 3000 (Invitrogen) according to standard protocols. Control cells received non-targeting siRNA (Sense: 5′-UUCUCCGAACGUGUCACGUTT-3'; Antisense: 5′-ACGUGACACGUUCGGAGAATT-3′).

### 2.7 Cell cycle analysis

Following 24-h serum starvation for cell cycle synchronization, the cells were re-fed with complete medium and incubated for an additional 24–36 h (HHL5 and PLC/PRF/5 cells for 24 h, and MHCC-97H cells for 36 h) prior to harvesting. Synchronized cells were fixed in 75% ethanol overnight, treated with RNase, and stained with propidium iodide using the Cell Cycle Assay-PI/RNase Staining Kit (Dojindo). DNA content analysis was performed on a BD LSRFortessa flow cytometer, with data processed using FlowJo software (v10.6.2).

### 2.8 Subcellular fractionation

Nuclear and cytoplasmic extracts were prepared using the Nuclear/Cytoplasmic Extraction Reagent Kit (Beyotime) supplemented with protease inhibitors. Protein localization was confirmed by immunoblotting using compartment-specific markers (Lamin B1 (12987-1-AP, Proteintech) for nuclear; GAPDH (AC033, ABclonal) for cytoplasmic fractions).

### 2.9 Western blotting

Total protein lysates were prepared using RIPA buffer (Beyotime) containing protease and phosphatase inhibitors. Protein concentration was determined by BCA assay (Thermo Fisher). Samples were separated by 10% SDS-PAGE, transferred to nitrocellulose membranes, and probed with primary antibodies (4°C, overnight) followed by appropriate secondary antibodies. The primary antibodies used in Western blotting included PPP1R12B antibody (13366-1-AP, Proteintech), PAK1 Polyclonal antibody (21401-1-AP), gamma-PAK2 antibody (sc-373740, Santa Cruz), Cyclin D1 antibody (2978, CST), beta-catenin antibody (37447, CST), Phospho-beta-catenin (Ser675) antibody (4176, CST), beta-actin antibody (AC004, ABclonal). The secondary antibodies used were IRDye 800CW Goat anti-Mouse IgG (H + L) and IRDye 800CW Goat anti-Rabbit IgG (H + L) from LI-COR. Detection was performed using an Odyssey Sa Imaging System (LI-COR).

### 2.10 Co-immunoprecipitation (Co-IP)

Cells were collected and lysed in IP buffer with protease inhibitors (Beyotime). Subsequently, Protein A/G Magnetic Beads (MCE) was added to total protein, incubated at 4°C for 1 h with mild rotation. After centrifugation at 12,000 rpm for 15 min, the harvested supernatant was incubated with 2 μg anti-FLAG (66008-4-IP, Proteintech) and 2 μg anti-IgG (sc-2025, Santa Cruz), or 2 μg anti-PAK2 antibody (19979-1-AP, Proteintech) and 2 μg anti-IgG (12–370, Sigma-Aldrich) at 4°C overnight with gentle rotation. A magnetic rack was used to recover the precipitated protein complex. Immune complexes were captured with magnetic beads, washed extensively, and analyzed by Western blotting. 10% of total protein was applied as input control.

### 2.11 Immunohistochemical (IHC) staining and IHC scores

Formalin-fixed, paraffin-embedded sections (3–5 μm) underwent antigen retrieval before incubation with PPP1R12B antibody (13366-1-AP, Proteintech) (4°C, overnight). Detection employed HRP-conjugated secondaries (Supervision) with DAB chromogen (DAKO). Slides were counterstained with hematoxylin, scanned (Leica Aperio AT2), and analyzed using Aperio ImageScope (v12.4.6) and ImageJ. Staining intensity (0: negative, 1: weak, 2: moderate, 3: strong) and positive cells distribution (0%–100%) were combined to generate H-scores (range 0–300) (Liu W. et al., 2015).

### 2.12 Survival analysis

Patients were stratified by PPP1R12B expression (low/medium/high) based on IHC scores. Combined with overall survival information of HCC patients, Kaplan-Meier survival curves were generated using GraphPad Prism 10, with statistical significance assessed by log-rank test.

### 2.13 Immunofluorescence

Cells fixed with 4% paraformaldehyde were permeabilized (0.1% Triton X-100), blocked with 2% BSA, and incubated with primary antibodies (4°C, overnight) followed by fluorophore-conjugated secondaries. The primary antibodies used in Immunofluorescence were same as in Western blotting, plus PAK2 antibody (19979-1-AP, Proteintech). The fluorophore-conjugated secondary antibodies were bought from Invitrogen (A-11008, A-11001, A-21428, and A-21422). Nuclei were counterstained with DAPI (Beyotime) before imaging on a Leica TCS SP8 confocal microscope.

### 2.14 β-Catenin transcriptional activity

TOPFlash/FOPFlash reporter assays were conducted by co-transfecting 250 ng of TOPFlash or FOPFlash reporter plasmid with 20 ng pRL-TK (Promega) using Lipofectamine 3000 (Invitrogen). Luciferase activity was measured 48 h post-transfection using the Dual-Luciferase Assay System (Promega). Data were normalized to Renilla luciferase activity, with the TOPFlash/FOPFlash ratio representing relative luciferase activity.

### 2.15 Tumor xenograft experiment

All animal procedures were approved by the Institutional Animal Care Committee at Eastern Hepatobiliary Surgery Hospital. Male BALB/c nude mice (4–5 weeks; GemPharmatech) were housed under specific pathogen-free conditions. Huh7 overexpression and control cells (5 × 10^6^) suspended in Matrigel/medium (3:2) were injected subcutaneously. Tumor dimensions were measured every 3 days, with volume calculated as 0.5 × length × width^2^. Mice were euthanized when tumors reached 1,500 mm^3^.

### 2.16 Phosphoproteomic sequencing analysis

Phosphoproteomic sequencing analysis was performed by Jingjie Biotechnology Co., Ltd. At first, protein samples lysed in urea buffer (8M urea, 1% protease/phosphatase inhibitors) via ultrasonication and precipitated with acetone, digested with trypsin (1:50 w/w), reduced (DTT), alkylated (IAA), and desalted (Strata X SPE). Next, phosphopeptides were enriched using IMAC microspheres with wash (50% ACN/0.5% acetic acid → 30% ACN/0.1% TFA) and elution (10% NH_4_OH) before LC-MS/MS analysis on an Orbitrap Exploris 480 mass spectrometer coupled to an EASY-nLC 1200 UPLC system. And then, data were processed using Spectronaut (v18) against the UniProt human database (Homo_sapiens_9606_SP_20231220).

### 2.17 Statistical analyses

Data analysis employed GraphPad Prism 10 and SPSS 22.0. Continuous variables are presented as mean ± SEM and compared using Student’s *t*-test. Categorical variables were analyzed by *χ* ^2^ test. Survival data were evaluated by Kaplan-Meier method with log-rank test. Correlation analyses used Spearman’s rank test. All tests were two-tailed with *α* = 0.05.

## 3 Results

### 3.1 PPP1R12B is downregulated and prognostic in HCC

**FIGURE 1:**
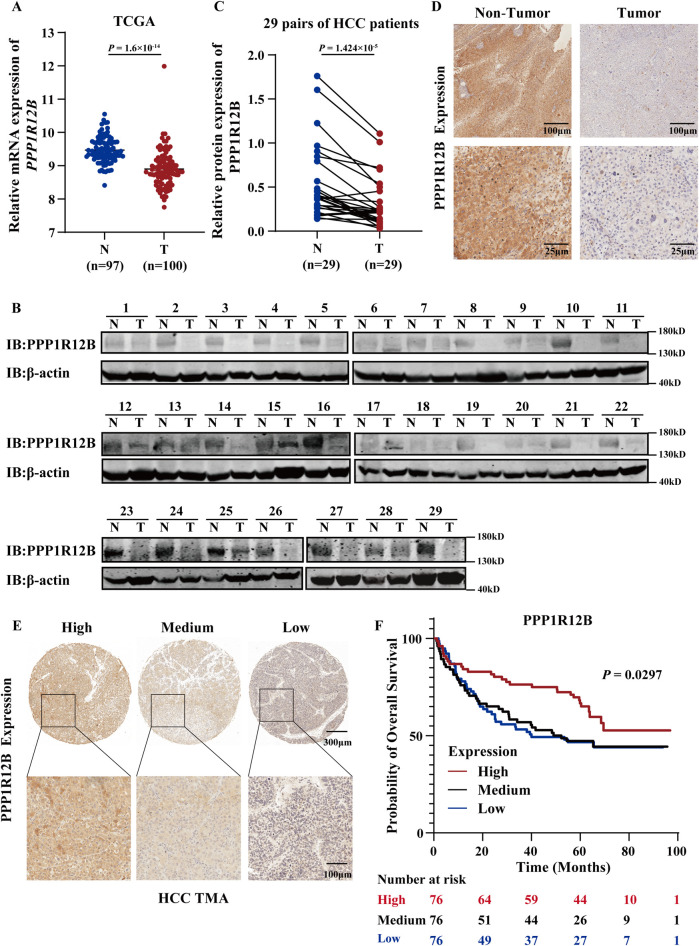
Clinical significance of PPP1R12B expression in HCC. **(A)** Comparative analysis of PPP1R12B transcript levels (log2 TPM) between HCC specimens (T, *n* = 100) and normal hepatic tissues (N, *n* = 97) from TCGA database (P = 1.6 × 10^−34^). **(B)** Immunoblot analysis confirming reduced PPP1R12B protein levels in HCC specimens compared to paired adjacent non-tumor tissues (*n* = 29). **(C)** Immunoblot analysis demonstrating differential PPP1R12B protein expression in 29 paired HCC tumors and adjacent non-tumor tissues (P = 1.424 × 10^−5^). Protein quantification was normalized to β-actin loading controls. **(D)** Immunohistochemical staining illustrating representative PPP1R12B expression patterns in matched tumor and adjacent non-tumor tissue sections. The scale bar = 100 μm and 25 μm. **(E)** Tissue microarray (TMA) analysis depicting heterogeneous PPP1R12B immunoreactivity across HCC clinical samples. The scale bar = 300 μm and 100 μm. **(F)** Kaplan-Meier survival curves demonstrating significantly prolonged overall survival in HCC patients with high PPP1R12B expression (*n* = 228, log-rank trend test).

The Cancer Genome Atlas (TCGA) datasets were used to assess PPP1R12B expression patterns. The results revealed significant downregulation of PPP1R12B mRNA in HCC tissues relative to adjacent non-tumor controls (Figure 1A). Western blot and immunohistochemistry (IHC) experiments of 29 paired samples revealed significantly lower PPP1R12B protein expression in HCC tumor tissues compared to matched adjacent non-tumor tissues (Figures 1B–D). Moreover, we subsequently performed survival analysis using HCC tissue microarray (TMA). All 228 tissue spots were individually scored by IHC and categorized into high (*n* = 76), medium (*n* = 76), and low (*n* = 76) PPP1R12B expression groups based on IHC scores (Figure 1E), followed by Kaplan-Meier survival curve analysis with clinical follow-up data. The survival analysis demonstrated that high PPP1R12B expression correlated with significantly better survival outcomes (Figure 1F). Hence the low and medium expression groups showed nearly identical survival patterns, we combined these two groups and then analyzed the differences in clinicopathological characteristics between the high versus low/medium expression groups. HCC patients with high PPP1R12B expression were apt to have higher age, recurrence-free survival time, overall survival time, and better final clinical outcome as shown in Table 1.

**TABLE 1:** Correlation between PPP1R12B expression and clinic characteristics.

Characteristic	PPP1R12B expression		*P*-value
	Low and Medium (*n* = 152)	High (*n* = 76)	
Age/years	52.36 ± 11.43	57.96 ± 11.45	0.000585
Gender/n (%)			
Male	126 (82.9)	66 (86.8)	0.441
Female	26 (17.1)	10 (13.2)	
Tumor size/cm	7.33 ± 3.96	6.66 ± 3.78	0.226
Tumor number*/n	1.09 ± 0.305	1.16 ± 0.402	0.139
RFS/month	33.71 ± 29.38	44.40 ± 27.76	0.009
RFS status/n (%)			
Alive	56 (36.8)	34 (44.7)	0.250
Dead	96 (63.2)	42 (55.3)	
OS/Month	41.49 ± 28.35	53.20 ± 25.58	0.002
OS Status/n (%)			
Alive	70 (46.1)	46 (60.5)	0.039
Dead	82 (53.9)	30 (39.5)	
Recurrence*/n (%)			
Absent	147 (97.4)	74 (97.4)	0.994
Present	4 (2.6)	2 (2.6)	
Liver Cirrhosis/n (%)			
Absent	72 (47.4)	29 (38.2)	0.187
Present	80 (52.6)	47 (61.8)	
HBV/n (%)			
Absent	4 (2.6)	6 (7.9)	0.067
Present	148 (97.4)	70 (92.1)	
HCV/n (%)			
Absent	150 (98.7)	73 (96.1)	0.201
Present	2 (1.3)	3 (3.9)	
TNM stage*/n (%)			
I	116 (77.9)	55 (73.33)	0.583
II	5 (3.35)	1 (1.33)	
III	5 (3.35)	3 (4.0)	
IV	23 (15.4)	16 (21.33)	
Tumor differentiation*/n (%)			
Poorly	5 (3.4)	1 (1.3)	0.509
Moderately	140 (95.9)	74 (98.7)	
Well	1 (0.7)	0	
Extrahepatic metastasis/n (%)			
Absent	131 (86.2)	60 (78.9)	0.162
Present	21 (13.8)	16 (21.1)	
Lymphatic metastasis/n (%)			
Absent	149 (98.0)	75 (98.7)	0.721
Present	3 (2.0)	1 (1.3)	
Bile duct thrombi/n (%)			
Absent	150 (98.7)	75 (98.7)	0.742
Present	2 (1.3)	1 (1.3)	
Vascular tumor emboli/n (%)			
Absent	124 (81.6)	65 (85.5)	0.456
Present	28 (18.4)	11 (14.5)	

* Data were not available for all patients.

Student’s *t*-test was used to compare continuous variables, and the data were presented as the mean ± SEM., The χ-square test and Fisher’s exact test was used to compare qualitative variables.

### 3.2 PPP1R12B represents an important protective factor in HCC patients

**FIGURE 2:**
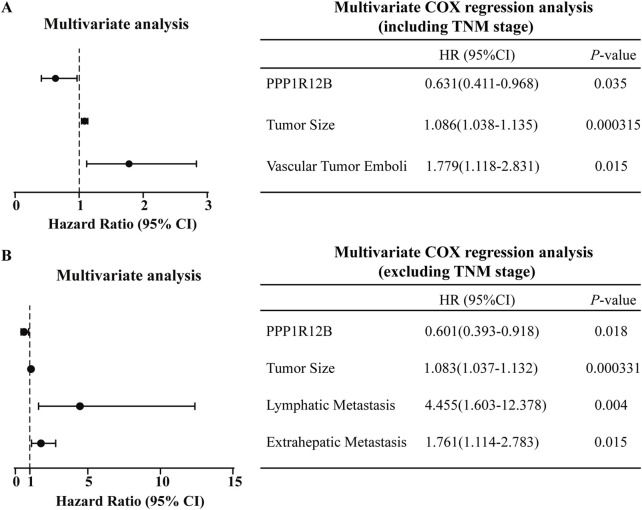
Prognostic significance of PPP1R12B in HCC patients. **(A)** Multivariate Cox regression analysis incorporating TNM staging identified tumor size (HR = 1.086, 95% CI 1.038–1.135, P = 0.000315) and vascular invasion (HR = 1.779, 95% CI 1.118–2.831, p = 0.015) as independent risk factors, while PPP1R12B expression (HR = 0.631, 95% CI 0.411–0.968, p = 0.035) demonstrated protective effects. **(B)** Alternative multivariate model excluding TNM staging confirmed PPP1R12B’s persistent protective role (HR = 0.601, 95% CI 0.393–0.918, p = 0.018), with tumor size (HR = 1.083, 95% CI 1.037–1.132, p = 0.000331), lymph node metastasis (HR = 4.455, 95% CI 1.603–12.378, p = 0.004), and distant metastasis (HR = 1.761, 95% CI 1.114–2.783, p = 0.015) emerging as additional risk factors. Both models were constructed using forward stepwise regression with AIC-based variable selection.

To determine whether PPP1R12B represents an independent prognostic factor in HCC patients, we then performed multivariate COX regression analysis based on the HCC TMA results and clinic characteristics data of HCC patients (Supplementary Table S1). Initially, univariate Cox analysis was used to identify insignificant prognostic factors which were excluded from further modeling (p ≥ 0.05). As results, the following factors were included in further multivariate analysis: PPP1R12B expression, tumor size, lymph node metastasis, distant metastasis, TNM stage, bile duct tumor thrombus, and vascular invasion. Given that TNM stage inherently incorporates data on lymph node metastasis and distant metastasis, we conducted separate statistical analyses for two scenarios: with and without TNM stage inclusion. Using forward stepwise regression to construct optimal models, we found that: When TNM stage was included as a covariate, tumor size and vascular invasion emerged as risk factors while PPP1R12B served as a protective factor (Figure 2A); and when TNM stage was excluded, PPP1R12B remained protective, while tumor size, lymph node metastasis and distant metastasis became significant risk factors (Figure 2B). These results collectively indicate that PPP1R12B represents an important protective factor in HCC patients.

### 3.3 PPP1R12B inhibits HCC cell proliferation

**FIGURE 3:**
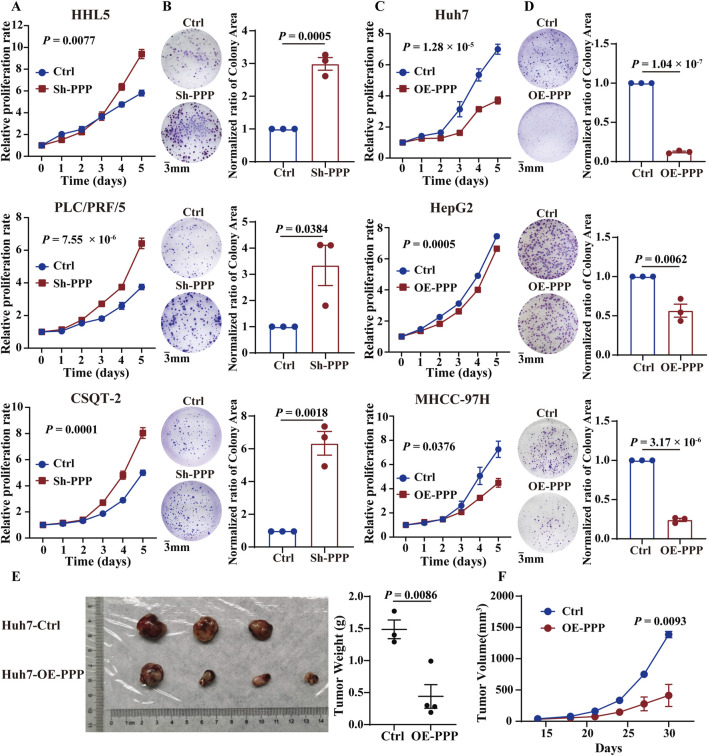
PPP1R12B inhibits HCC cell proliferation *in vivo* and *in vitro*. **(A)** Cell viability analysis demonstrating enhanced proliferation in PPP1R12B-knockdown HCC cells compared to controls (HHL5-P = 0.0077, PLC/PRF/5-P = 7.55 × 10^−6^, CSQT-2-P = 0.0001). **(B)** Clonogenic survival assays showing increased colony formation capacity following PPP1R12B depletion. Quantitative data represent mean colony counts from three independent experiments (±SEM) (HHL5-P = 0.0005, PLC/PRF/5-P = 0.0384, CSQT-2-P = 0.0018). **(C)** Cell viability analysis revealing significant proliferation inhibition in PPP1R12B-overexpressing HCC cells versus vector controls (Huh7-P = 1.28 × 10^−5^, HepG2-P = 0.0005, MHCC-97H-P = 0.0376). **(D)** Representative images and quantification of colony formation assays demonstrating reduced proliferative capacity in PPP1R12B-overexpressing cells (mean ± SEM) (Huh7-P = 1.04 × 10^−7^, HepG2-P = 0.0062, MHCC-97H-P = 3.17 × 10^−6^). **(E)** Comparative tumor weights from xenograft models at endpoint, showing significant reduction in PPP1R12B-overexpressing Huh7 cell-derived tumors versus controls (P = 0.0086). **(F)** Longitudinal tumor growth kinetics in nude mice implanted with PPP1R12B-modified Huh7 cells. Data points represent mean tumor volumes (±SEM) measured every 3 days (P = 0.0093).

Based on the baseline expression levels of PPP1R12B across hepatocellular carcinoma cell lines (Supplementary Figure S1A), we constructed stable PPP1R12B-overexpressing cell lines in Huh7, HepG2, and MHCC-97H (with relatively low natural expression), and stable knockdown cell lines in PLC/PRF/5 and CSQT-2 (with relatively high endogenous expression). Besides, human normal liver cell lines HHL5 was also constructed as a PPP1R12B knockdown cell lines. The CCK-8 assays and plate colony formation experiment demonstrated that PPP1R12B knockdown significantly enhanced proliferative capacity, whereas overexpression of PPP1R12B suppressed cell proliferation (Figures 3A–D). Using PPP1R12B-overexpressing Huh7 cells and controls cells, we performed xenograft tumor experiments in nude mice, periodically measuring tumor volume to compare growth rates between groups. Xenograft experiments and quantitative analysis revealed a significant reduction in mean tumor weight in PPP1R12B-overexpressing mice relative to control groups (Figure 3E). Meanwhile, each inoculation including Huh7 cells with PPP1R12B-overexpression developed into a slowly growing and small tumor than controls (Figure 3F). These overexpression and knockdown results suggested PPP1R12B might function as tumor suppressor in HCC cell proliferation.

### 3.4 PPP1R12B induces cell cycle arrest at the G0/G1 to S phase

**FIGURE 4:**
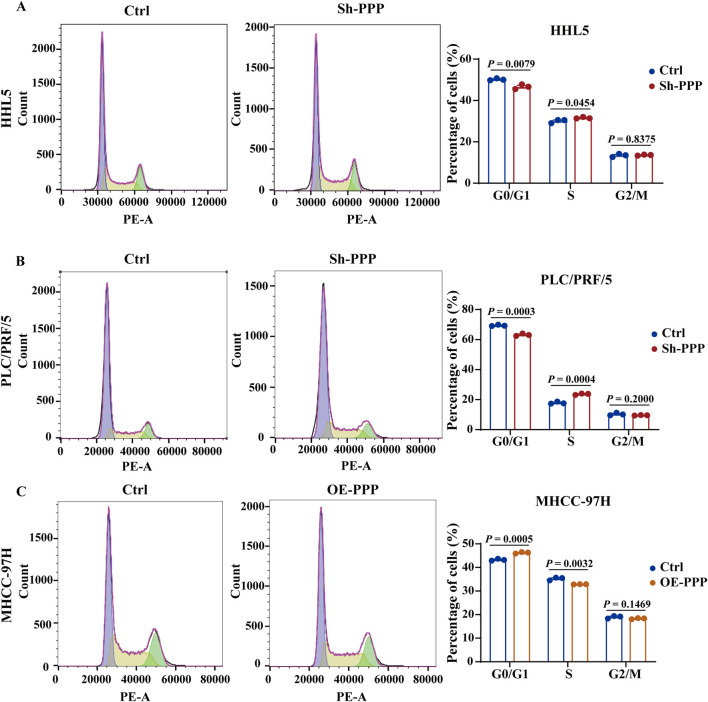
PPP1R12B induces cell cycle arrest at the G0/G1 to S phase. **(A)** Flow cytometric analysis of cell cycle distribution in HHL5 cells following PPP1R12B knockdown, demonstrating a significant decrease in G0/G1 phase population (50.2% vs. 46.6% in controls, P = 0.0079) and concomitant increase in S phase cells (30.0% vs. 31.6%, P = 0.0454). **(B)** Flow cytometric analysis of cell cycle distribution in HHL5 cells and PLC/PRF/5 cells following PPP1R12B knockdown, demonstrating a significant decrease in G0/G1 phase population (69.4% vs. 63.1% in controls, P = 0.0003) and concomitant increase in S phase cells (17.8% vs. 23.7%, P = 0.0004). **(C)** Cell cycle profiling of MHCC-97H cells overexpressing PPP1R12B revealed G0/G1 phase arrest (43.3% vs. 46.2% in vector controls, P = 0.0005) with reduced S phase entry (35.2% vs. 32.9%, P = 0.0032). Data represent mean percentages (±SEM) from three independent experiments.

Given the well-established relationship between proliferation and cell cycle regulation, we hypothesized that PPP1R12B might influence HCC cell proliferation by modulating the cell cycle. To further investigate this, PPP1R12B overexpressing MHCC-97H cells and downregulated PLC/PRF/5 and HHL5 cells was chosen for cell cycle analysis by flow cytometry. After serum starvation for cell cycle synchronization and an additional 24–36 h incubation, flow cytometry analysis revealed that altered PPP1R12B expression indeed affected HCC cell cycle distribution, primarily impacting the G0/G1 and S phases. More specifically, PPP1R12B knockdown accelerated progression from G0/G1 to S phase (Figures 4A,B), whereas overexpression caused cell cycle arrest at the G0/G1-S transition boundary (Figure 4C).

### 3.5 PAK2 serves as a crucial regulator in PPP1R12B-mediated HCC proliferation suppression

**FIGURE 5:**
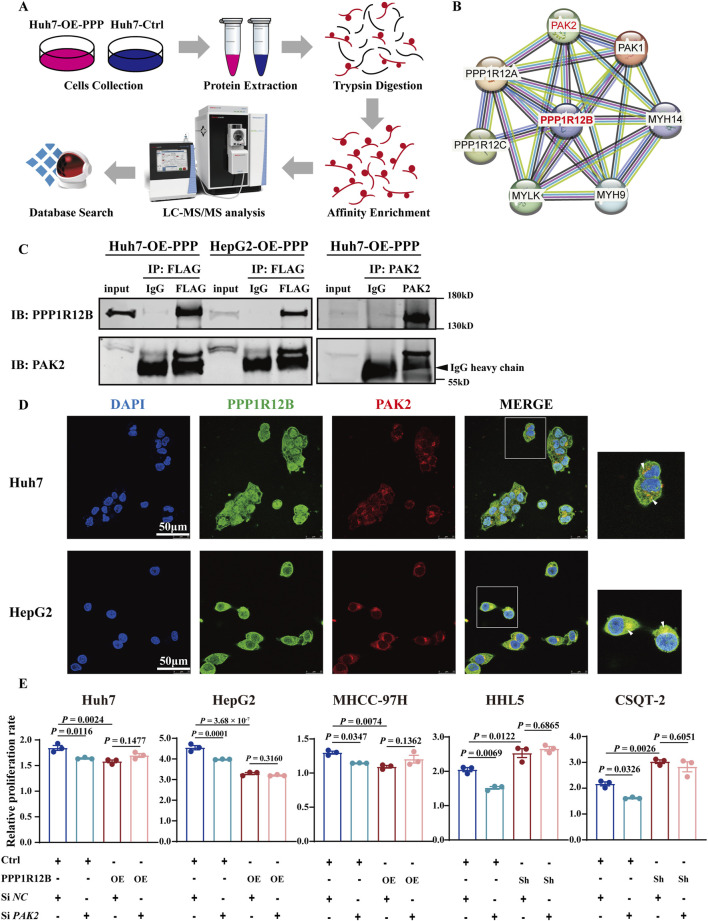
PAK2 plays a key role in PPP1R12B-mediated HCC proliferation suppression. **(A)** Schematic overview of the phosphoproteomic profiling strategy comparing PPP1R12B-overexpressing Huh7 cells with vector controls. The workflow includes protein extraction, tryptic digestion, phosphopeptide enrichment, LC-MS/MS analysis, and database search. **(B)** Protein interaction network of differentially phosphorylated proteins, with PPP1R12B positioned as a central node. The network was constructed using STRING with a confidence score threshold of 0.7. **(C)** Co-immunoprecipitation analysis demonstrating physical interaction between PPP1R12B and PAK2. Left: Flag-tagged PPP1R12B immunoprecipitated endogenous PAK2 in both Huh7 and HepG2 overexpression cells. Right: Reciprocal co-IP confirmed the interaction using PAK2 antibody for pulldown. **(D)** Immunofluorescence microscopy revealing subcellular co-localization of PPP1R12B (green) and PAK2 (red) in HCC cells. Nuclei were counterstained with DAPI (blue). Scale bars: 50 μm. **(E)** Functional rescue experiments showing that PAK2 knockdown (siPAK2) abrogated the proliferative effects of PPP1R12B modulation in CCK-8 assays.

Phosphoproteomic sequencing analysis using PPP1R12B-overexpressing Huh7 cells and controls was performed to identify the critical molecules involved in PPP1R12B-mediated HCC proliferation suppression (Figure 5A). Combined with protein interaction network analysis, the potential interaction between PPP1R12B and PAK1/PAK2 were found (Figure 5B). Then we examined the protein expression levels of both PAK1 and PAK2 in PPP1R12B-overexpressing Huh7 and MHCC-97H cell lines. Western blot analysis revealed that PAK1 expression remained relatively unchanged, whereas PAK2 expression showed a significant decrease (Fig. S1B). Consequently, we selected PAK2 as the focus for subsequent investigations.

We first confirmed this interaction through co-immunoprecipitation (Co-IP) using Flag antibodies in Flag-tagged Huh7 and HepG2 overexpression cell lines (Figure 5C). Subsequent reciprocal Co-IP with PAK2 antibodies in Huh7 overexpression cells further verified the PPP1R12B-PAK2 interaction (Figure 5C). Moreover, confocal microscopy clearly demonstrated co-localization of PAK2 and PPP1R12B in Huh7 and HepG2 cells, providing additional evidence for their interaction (Figure 5D).

Having established this interaction, we investigated whether PAK2 involves PPP1R12B-mediated HCC proliferation suppression. Using siRNA to knockdown PAK2 in PPP1R12B-overexpressing (Huh7, HepG2, MHCC-97H) and -knockdown (HHL5, CSQT-2) cell lines, we observed that PAK2 knockdown attenuated the proliferative changes induced by PPP1R12B modulation (Figure 5E). Control experiments showed that PAK2 positively regulated HCC cell proliferation, as its knockdown reduced proliferative capacity of HCC cells. These findings suggested that PPP1R12B likely exerts its effects on proliferation through PAK2.

### 3.6 PPP1R12B suppresses proliferation via the PAK2/β-catenin/cyclin D1 axis

**FIGURE 6:**
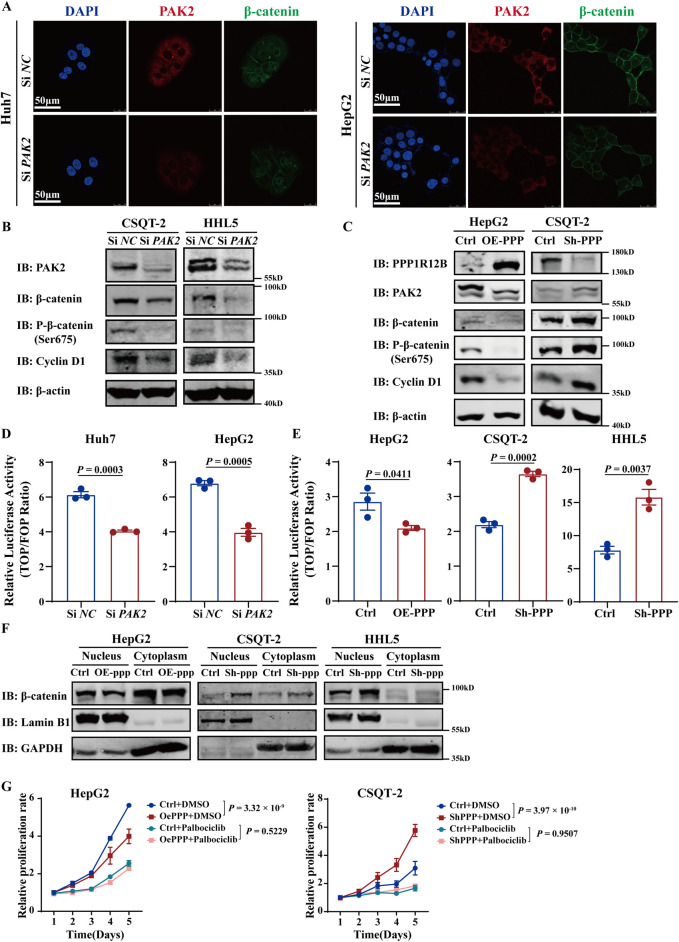
PPP1R12B suppresses proliferation via the PAK2/β-catenin/Cyclin D1 axis. **(A)** Confocal microscopy analysis demonstrating reduced β-catenin expression (green) following PAK2 (red) knockdown in Huh7 and HepG2 cells. Nuclei were counterstained with DAPI (blue). Scale bars: 50 μm. **(B)** PAK2-knockdown reduced total β-catenin, p-β-catenin (Ser675), and the expression of Cyclin D1 in CSQT-2 and HHL5 cells. **(C)** PPP1R12B overexpression decreased while knockdown increased the expression of PAK2, β-catenin, p-β-catenin (Ser675) and Cyclin D1 in HepG2 overexpression cells and CSQT-2 knockdown cells. **(D,E)** TOPFlash reporter assays measuring β-catenin transcriptional activity: **(D)** PAK2 knockdown significantly reduced β-catenin-mediated transcription (Huh7-P = 0.0003, HepG2-P = 0.0005); **(E)** PPP1R12B modulation correspondingly altered β-catenin activity (HepG2-P = 0.0411, CSQT-2-P = 0.0002, HHL5-P = 0.0037). **(F)** Subcellular fractionation analysis demonstrating PPP1R12B knockdown increased nuclear β-catenin accumulation. GAPDH and Lamin B1 served as compartment-specific controls. **(G)** CCK-8 assay revealed that palbociclib inhibited HCC cell proliferation and counteracted the proliferative changes induced by PPP1R12B modulation in HepG2 overexpression cells and CSQT-2 knockdown cells. The data were presented as mean ± SEM.

To explore PPP1R12B and PAK2 how to regulate HCC proliferation, we re-analysed the phosphoproteomic sequencing data focusing on PAK2. The results revealed its potential association with CTNNB1 (β-catenin) and SRC. Furthermore, the phosphoproteomic sequencing data indicated phosphorylation at β-catenin Ser675, with decreased levels, and phosphorylation at SRC Ser17, with increased levels (Supplementary Figure S1C). However, Western blot analysis in PPP1R12B-overexpressing Huh7 cells revealed no significant alterations in either total SRC protein levels or its phosphorylation status (Supplementary Figure S1D). Consequently, we prioritized β-catenin for subsequent mechanistic investigations. Reduced β-catenin expression following PAK2 knockdown was confirmed by confocal microscopy (Figure 6A). Since Ser675 phosphorylation was reported to enhance β-catenin stability and transcriptional activity (Zhu et al., 2012), we examined β-catenin activity after PAK2 knockdown. Indeed, β-catenin activity was significantly lower in PAK2-knockdown cells compared to controls (Figure 6D). We further demonstrated that PAK2 knockdown reduced total β-catenin, p-β-catenin (Ser675), and the expression of *CCND1* (a canonical β-catenin downstream gene) in CSQT-2 and HHL5 cells (Figure 6B). These findings suggested that PAK2 promotes β-catenin expression, Ser675 phosphorylation, and nuclear translocation.

Next, we investigated whether PPP1R12B similarly affects β-catenin localization. In CSQT-2 knockdown cells and HepG2 overexpression cells, PPP1R12B overexpression decreased while knockdown increased the expression of PAK2, β-catenin, p-β-catenin (Ser675) and *CCND1* (Figure 6C). β-catenin activity assays showed corresponding changes, with increased activity in knockdown cells and decreased activity in overexpression cells (Figure 6E). Nuclear-cytoplasmic fractionation experiment revealed that nuclear β-catenin levels were elevated in knockdown cells but reduced in overexpression cells compared to controls cells (Figure 6F). These results indicate that PPP1R12B inhibits β-catenin expression and Ser675 phosphorylation, thereby suppressing nuclear translocation and downstream *CCND1* expression.

Cyclin D1 (encoded by *CCND1*) forms complexes with cyclin-dependent kinases 4/6 (CDK4/6) to critically regulate cell cycle progression in G0/G1 phase (Bertoli et al., 2013). Palbociclib, an oral, reversible, selective CDK4/6 inhibitor, was used to test whether CDK4/6-Cyclin D complexes mediate PPP1R12B-mediated HCC proliferation suppression. We found that palbociclib inhibited HCC cell proliferation and counteracted the proliferative changes induced by PPP1R12B modulation (Figure 6G). This indirectly supports that PPP1R12B regulates HCC cell proliferation by affecting Cyclin D1 expression and subsequent cell cycle control.

### 3.7 Clinical correlation between PPP1R12B and PAK2/β-catenin

**FIGURE 7:**
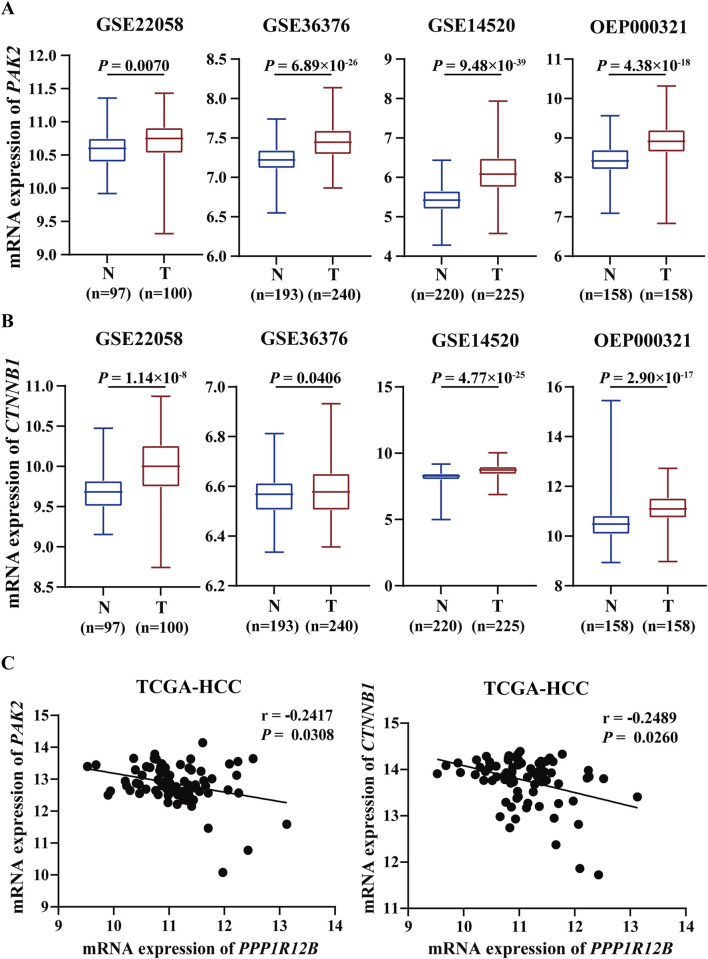
Clinical correlation between PPP1R12B and PAK2/β-catenin in HCC patients. **(A)** Transcriptomic analysis of PAK2 expression across multiple HCC cohorts (GSE22058, GSE36376, GSE14520, OEP000321) demonstrating significant upregulation in tumor tissues versus adjacent non-tumor controls (GSE22058-P = 0.0070, GSE36376-P = 6.89 × 10^−26^, GSE14520-P = 9.48 × 10^−39^, OEP000321-P = 4.38 × 10^−18^). Boxplots represent median values with interquartile ranges. Data presented as log2-transformed TPM values. **(B)** Comparative analysis of CTNNB1 (β-catenin) mRNA levels showing consistent overexpression in HCC specimens across above datasets (GSE22058-P = 1.14 × 10^−8^, GSE36376-P = 0.0406, GSE14520-P = 4.77 × 10^−25^, OEP000321-P = 2.90 × 10^−17^). Boxplots represent median values with interquartile ranges. Data presented as log2-transformed TPM values. **(C)** Spearman correlation analysis of TCGA-LIHC data revealing significant inverse relationships: PPP1R12B vs. PAK2: r = −0.2417, P = 0.0308; PPP1R12B vs. CTNNB1: r = −0.2489, p = 0.0260.

TCGA HCC cohort data was analyzed to further explore PAK2 and β-catenin expression patterns and prognostic significance. Across four HCC datasets (GSE22058, GSE36376, GSE14520, OEP000321), both PAK2 and β-catenin showed significantly higher expression in tumor tissues versus adjacent non-tumor tissues (Figures 7A,B). Correlation analysis of TCGA-HCC data demonstrated inverse relationships between PPP1R12B and both PAK2/β-catenin expression in HCC tissues (Figure 7C). Combined with our own findings on PPP1R12B expression pattern and prognostic significance, these results showed that PAK2 and β-catenin exhibit opposite expression patterns and prognostic associations compared to PPP1R12B, further supporting our proposed hypothesis.

## 4 Discussion

**FIGURE 8:**
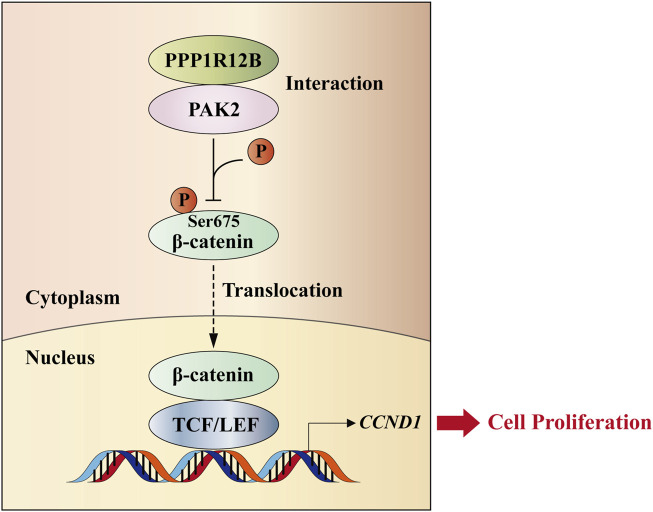
Schematic model. PPP1R12B suppresses HCC cell proliferation through the PAK2/β-catenin/Cyclin D1 axis. PPP1R12B could interact with PAK2 to suppress the expression and Ser675 phosphorylation of β-catenin, thereby inhibiting its nuclear translocation and the expression of its downstream target gene *CCND1*, which regulated cell proliferation.

To date, researches about PPP1R12B remain relatively scarce, with virtually no studies specifically investigating its role in HCC. In other cancer types, PPP1R12B exhibits contradictory functions. For example, PPP1R12B has been demonstrated to act as a tumor suppressor in colorectal cancer (Zhou et al., 2008; Ding et al., 2019; Tan et al., 2022) and esophageal carcinoma (Chen et al., 2022), while paradoxically serving as an oncogenic factor in Wilms’ tumor (He et al., 2021) and breast cancer (Fokkelman et al., 2016). Our previous work has confirmed that PPP1R12B suppresses proliferation, migration, invasion, and self-renewal of HCC cells (Chen et al., 2024). This study provides mechanistic insights by demonstrating that PPP1R12B inhibits HCC cell proliferation through the PAK2/β-catenin/Cyclin D1 axis (Figure 8). Given that PPP1R12B expression levels showing significant prognostic value for HCC patients, warranting further investigation.

Although the precise mechanisms require elucidation, PAK2 has been well-documented to be overexpressed or hyperactivated in various cancer types (Chen et al., 2025). Previous studies have identified a strong correlation between PAK2 overexpression and poor prognosis in HCC (Sato et al., 2013). In the present study, while we have definitively established the protein interaction between PPP1R12B and PAK2, several mechanistic questions remain unresolved: (1) whether this interaction inhibits PAK2 autophosphorylation; (2) the molecular mechanism by which PAK2 acquires kinase activity to phosphorylate β-catenin at Ser675 following PPP1R12B knockdown; and (3) the specific protein domains mediating this interaction. We propose a plausible hypothesis: During HCC pathogenesis, auto-phosphorylated PAK2 becomes activated. The PPP1R12B-PAK2 interaction may facilitate PP1-mediated dephosphorylation and consequent inactivation of PAK2. When PPP1R12B is knocked down, PP1 fails to recognize and dephosphorylate PAK2, allowing PAK2 to maintain its kinase activity, phosphorylate β-catenin at Ser675, promote β-catenin nuclear translocation, and enhance transcription of downstream targets like *CCND1*, ultimately leading to increased proliferation. These hypotheses and remaining questions demand systematic experimental validation in future studies.

Clinical evidence indicates that palbociclib exhibits excellent tolerability without significant hepatotoxicity (Turner et al., 2015), positioning it as an attractive candidate for liver cancer treatment. Several studies have demonstrated that palbociclib-based combination therapies represent promising novel strategies for HCC treatment (Sheng et al., 2021; Wang et al., 2024). Mechanistically, this highly specific CDK4/6 inhibitor functions by disrupting CDK4/6-Cyclin D complex formation and subsequent cell cycle progression. Our findings showed that palbociclib can counteract the proliferative effects induced by PPP1R12B modulation and confirm Cyclin D1 as a downstream target of PPP1R12B regulation. However, the potential involvement of CDK4/6 and other Cyclin D isoforms (D2/D3) in this regulatory network, as well as their specific contributions, remain to be elucidated in future investigations.

We observed heterogeneous PPP1R12B expression across HCC cell lines, potentially attributable to distinct cellular origins, or differential activation of compensatory pathways in different lines. Notably, normal liver cell lines HHL5 exhibited similar or even superior proliferative capacity to certain HCC cell lines (PLC/PRF/5 and CSQT-2), suggesting that conventional 2D monolayer cultures may incompletely recapitulate *in vivo* tumor dynamics. Furthermore, the relationship between PPP1R12B dysregulation and aberrant liver regeneration remains unresolved. These are limitations of this study.

In summary, our study delineates a previously unrecognized tumor-suppressive pathway in HCC proliferation, linking PPP1R12B to cell cycle control via PAK2/β-catenin/Cyclin D1 axis. At the molecular level, we have: (1) identified PPP1R12B as a novel PAK2-interacting protein; and (2) revealed that PPP1R12B inhibits these processes by suppressing β-catenin expression and Ser675 phosphorylation, ultimately leading to cell cycle arrest at the G0/G1 to S phase transition in HCC cells as well as in liver normal cells. This finding significantly strengthens the oncogenic role of PPP1R12B dysregulation, suggesting its function as a gatekeeper of cell cycle progression across both normal and malignant hepatic contexts. This means PPP1R12B may function as a “safety brake” in normal hepatocytes, while its absence triggers malignant transformation, and abnormal cell cycle in normal hepatocytes may create pro-tumorigenic microenvironment. These findings establish a previously unrecognized tumor-suppressive mechanism in HCC and highlight the therapeutic potential of targeting this signaling axis.

## Data Availability

The original data of quantitative phosphoproteomic analysis presented in the study are publicly available. This data can be found in iProX database with Project ID: IPX0012198000.

## Supplementary material

SUPPLEMENTARY FIGURE 1

**(A)** Baseline expression levels of PPP1R12B across hepatocellular carcinoma cell lines. **(B)** Western blot analysis revealed that PAK1 expression remained relatively unchanged, whereas PAK2 expression showed a significant decrease. **(C)** Phosphoproteomic sequencing data indicated phosphorylation at β-catenin Ser675, with decreased levels, and phosphorylation at SRC Ser17, with increased levels. **(D)** Western blot analysis in PPP1R12B-overexpressing Huh7 cells revealed no significant alterations in either total SRC protein levels or its phosphorylation status. The band was incubated with P-SRC (Ser17) antibody and stripped before being re-probed with the SRC antibody.
