# Supplementary figures and images for: PPP1R12B inhibits cell proliferation by inducing G0/G1 phase arrest via PAK2/β-catenin axis in hepatocellular carcinoma
Source: Front Cell Dev Biol. 2025 Jun 19;13:1621705. doi: 10.3389/fcell.2025.1621705 (PMC12222077; doi:10.3389/fcell.2025.1621705)

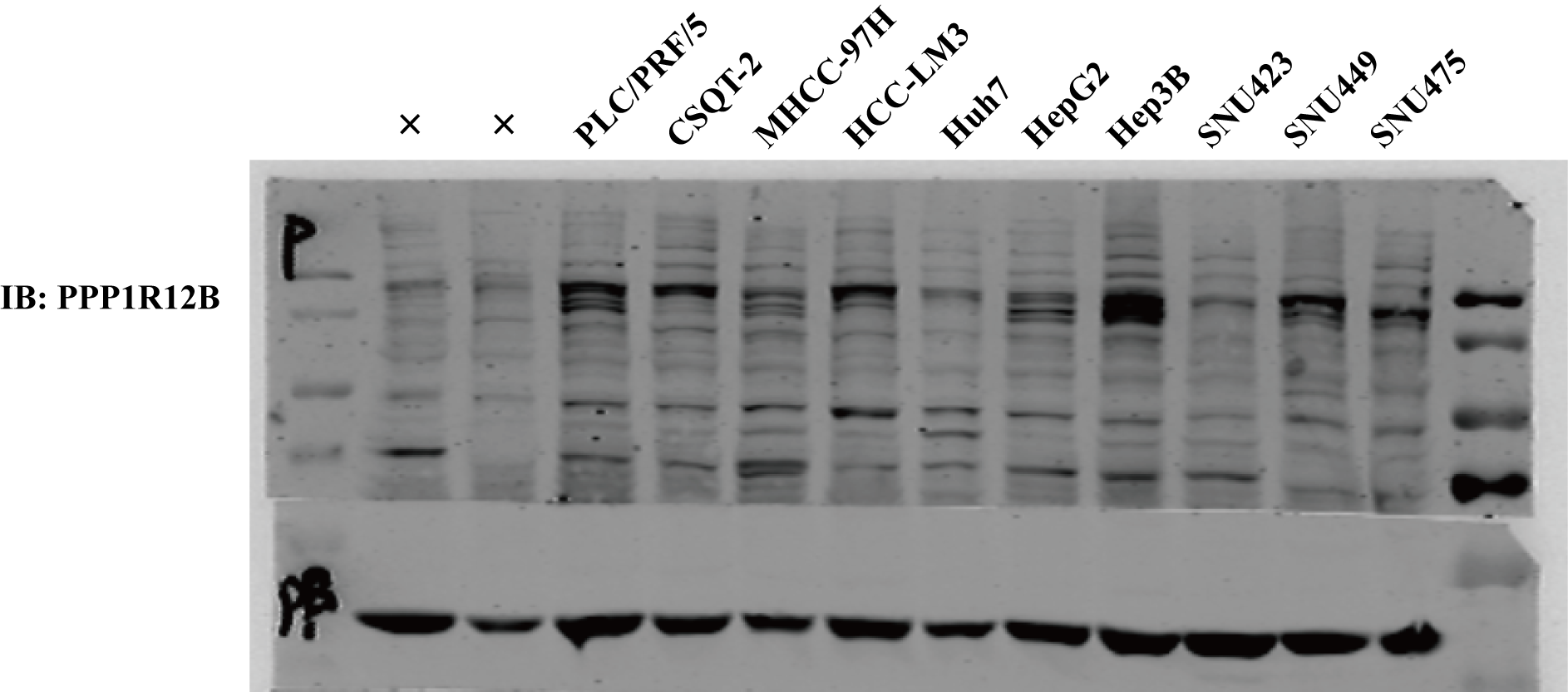

Supplement: Supplementary file 1 [file DataSheet1.zip › Supplementary file 3/WB images new/FigS1A-PPP1R12B.tif]

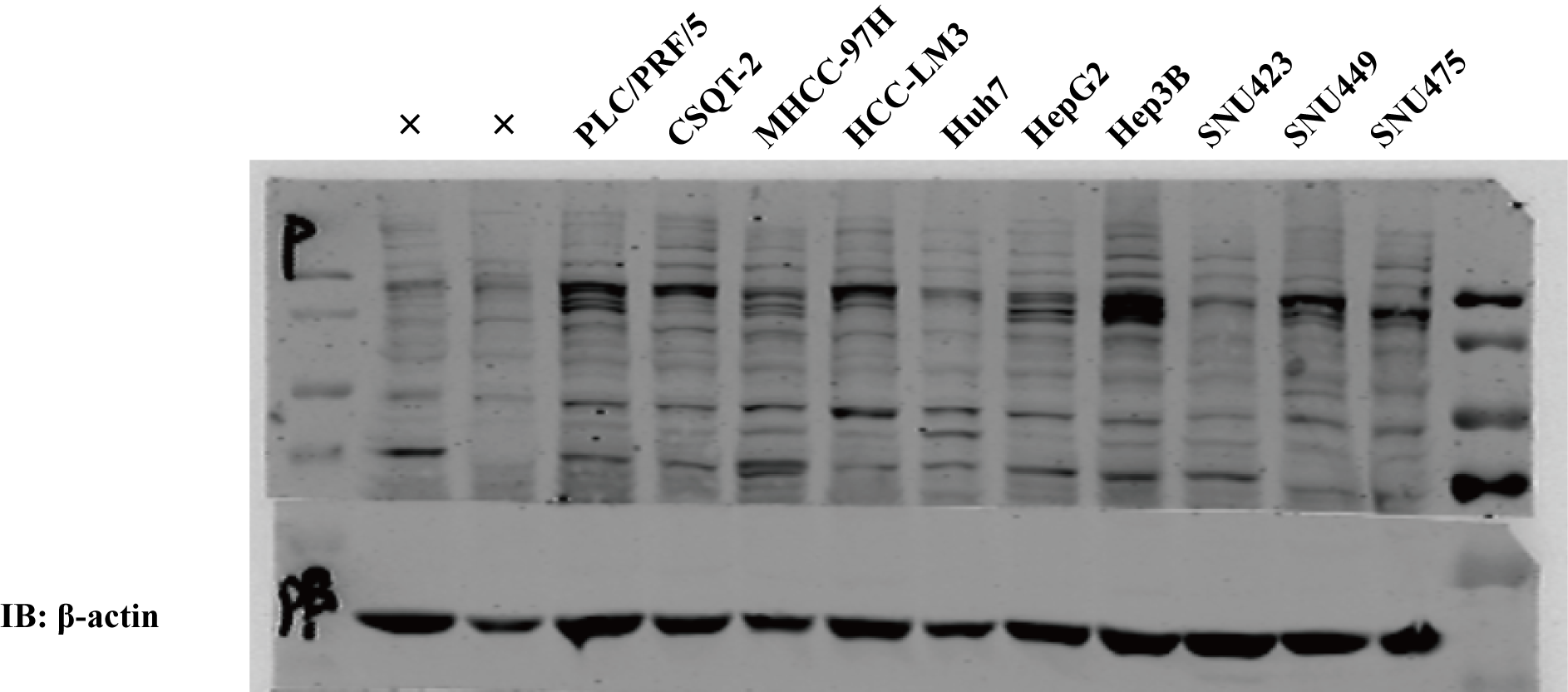

Supplement: Supplementary file 1 [file DataSheet1.zip › Supplementary file 3/WB images new/FigS1A-β-actin.tif]

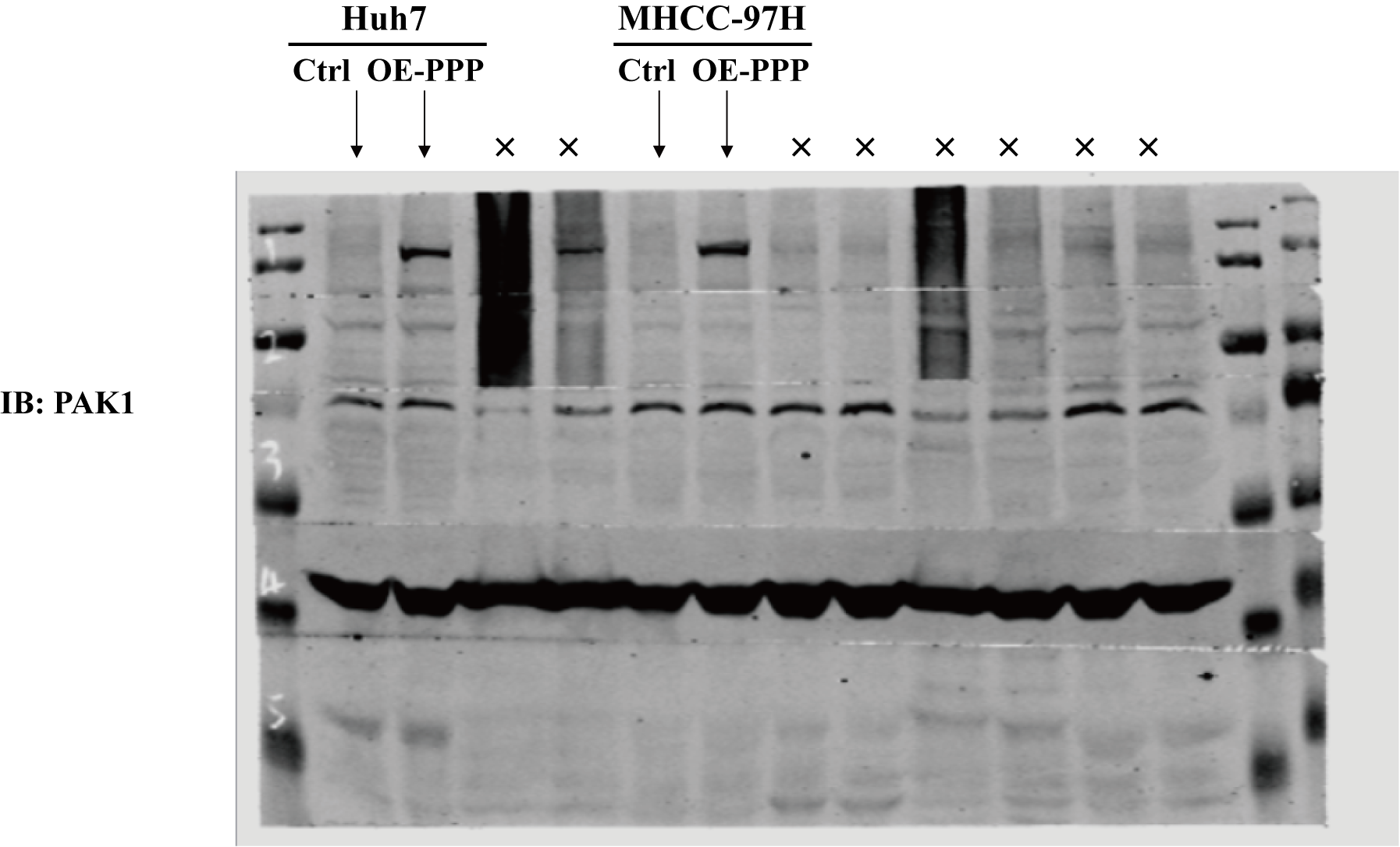

Supplement: Supplementary file 1 [file DataSheet1.zip › Supplementary file 3/WB images new/FigS1B-PAK1.tif]

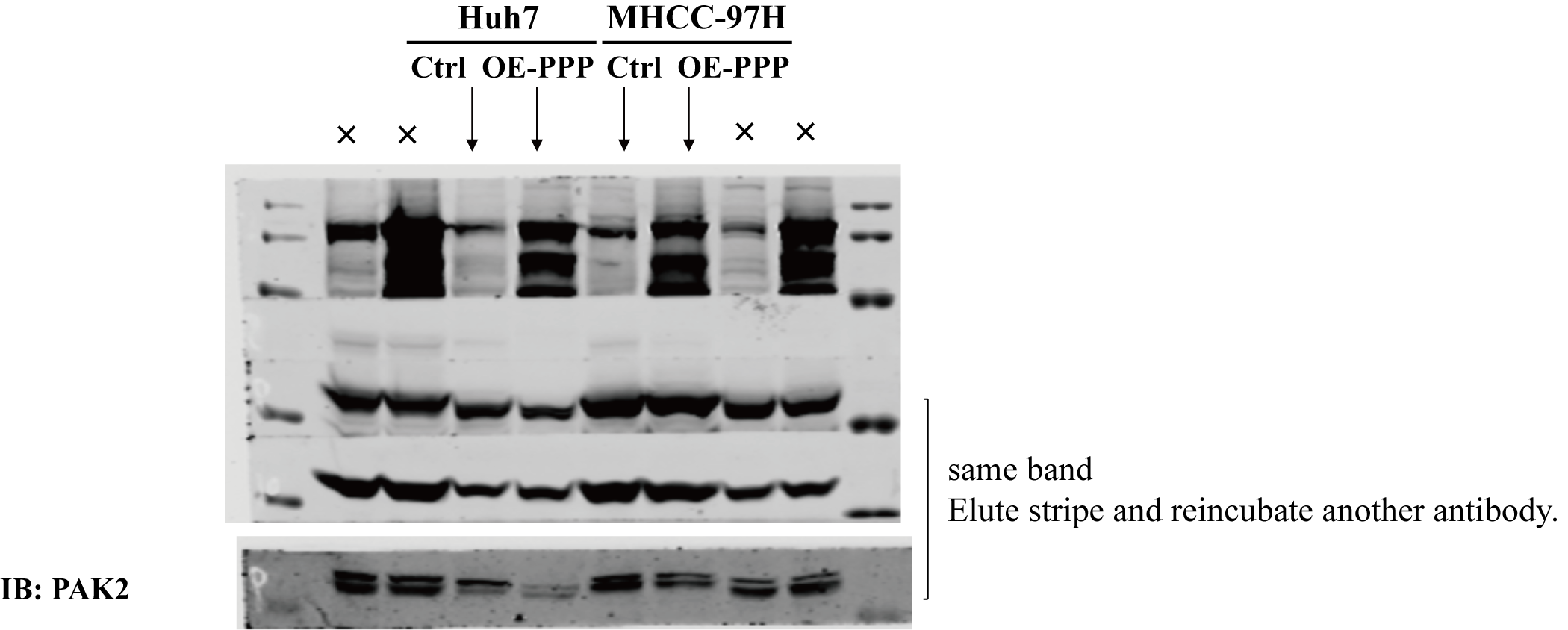

Supplement: Supplementary file 1 [file DataSheet1.zip › Supplementary file 3/WB images new/FigS1B-PAK2.tif]

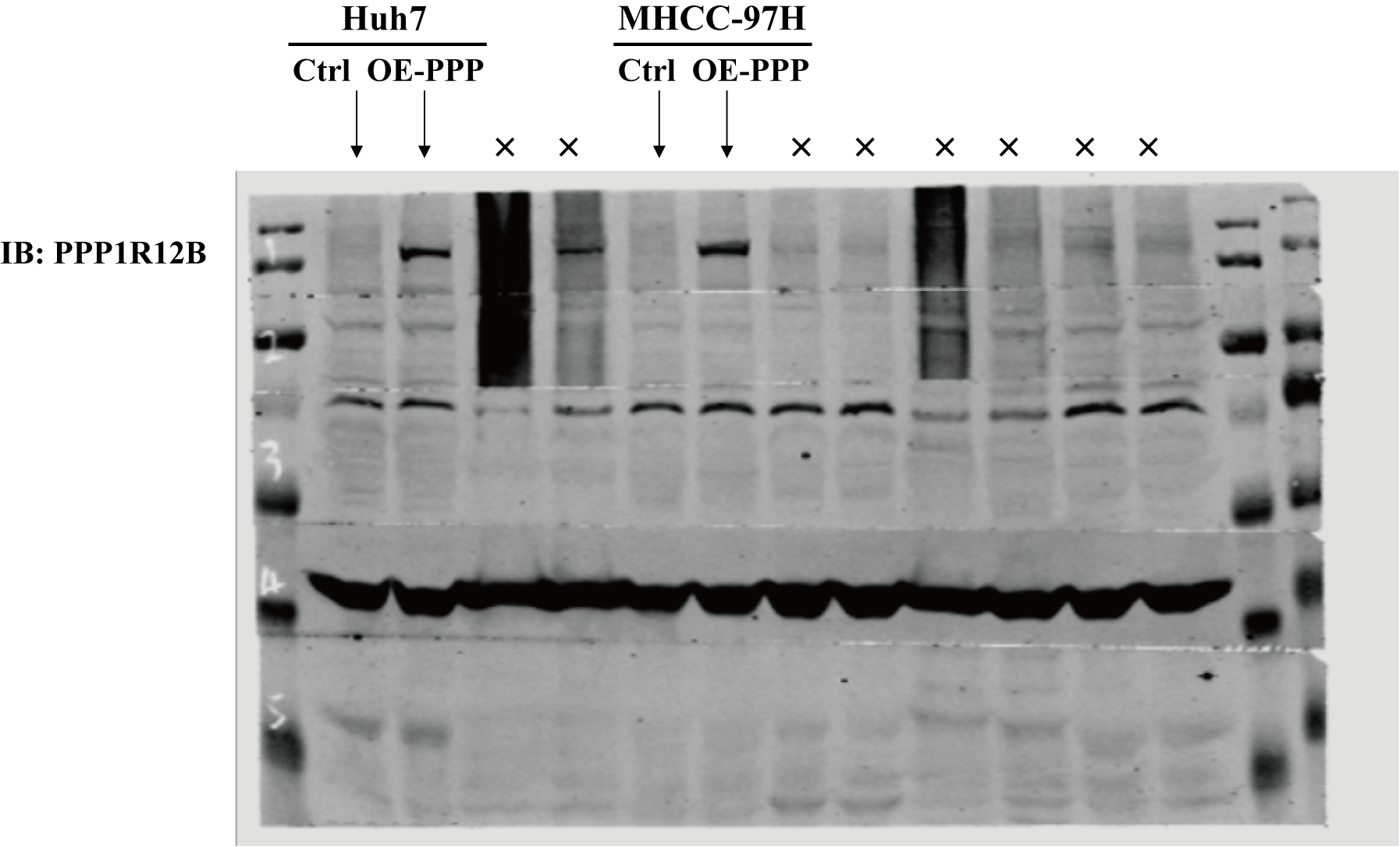

Supplement: Supplementary file 1 [file DataSheet1.zip › Supplementary file 3/WB images new/FigS1B-PPP1R12B(PAK1).tif]

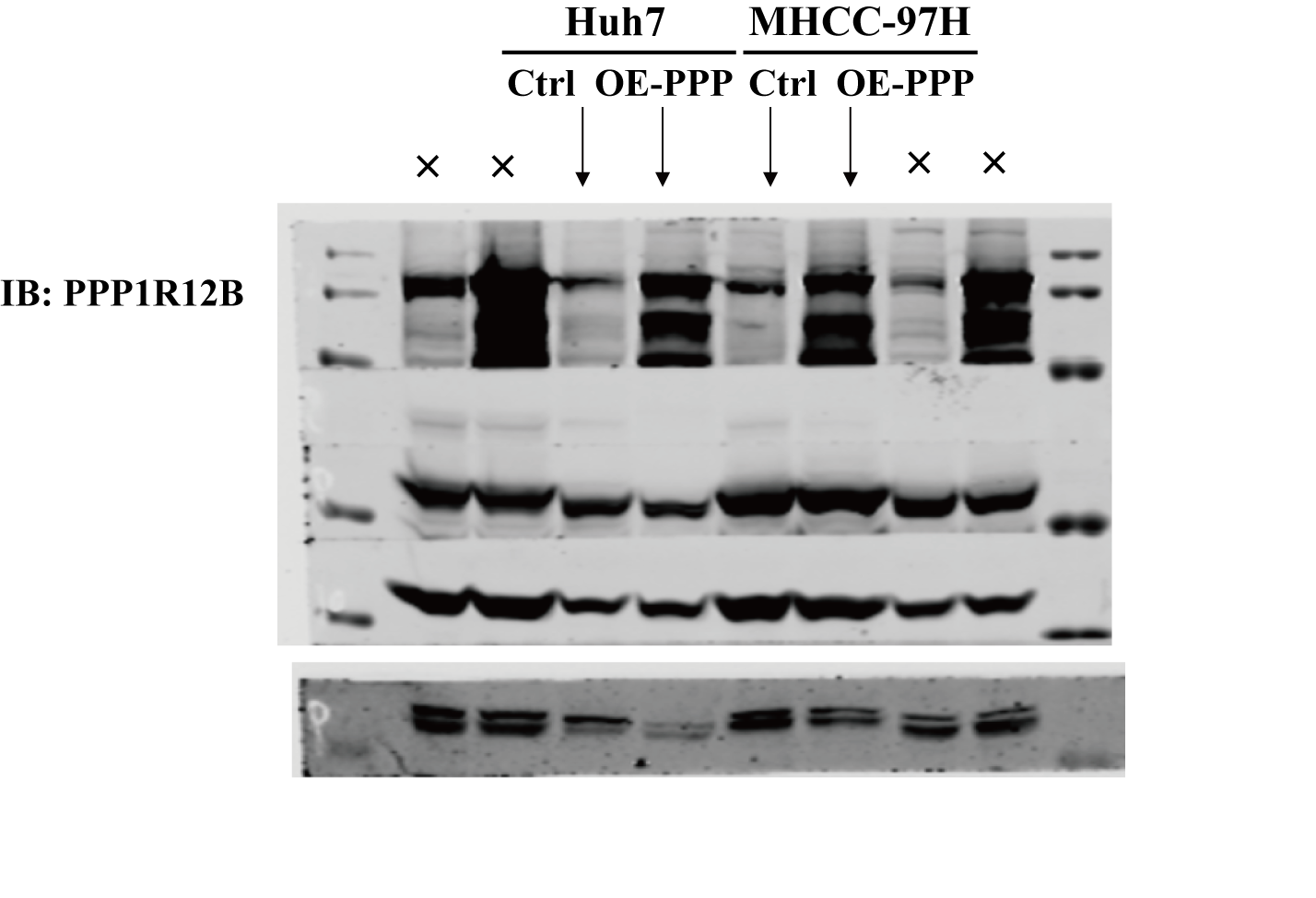

Supplement: Supplementary file 1 [file DataSheet1.zip › Supplementary file 3/WB images new/FigS1B-PPP1R12B(PAK2).tif]

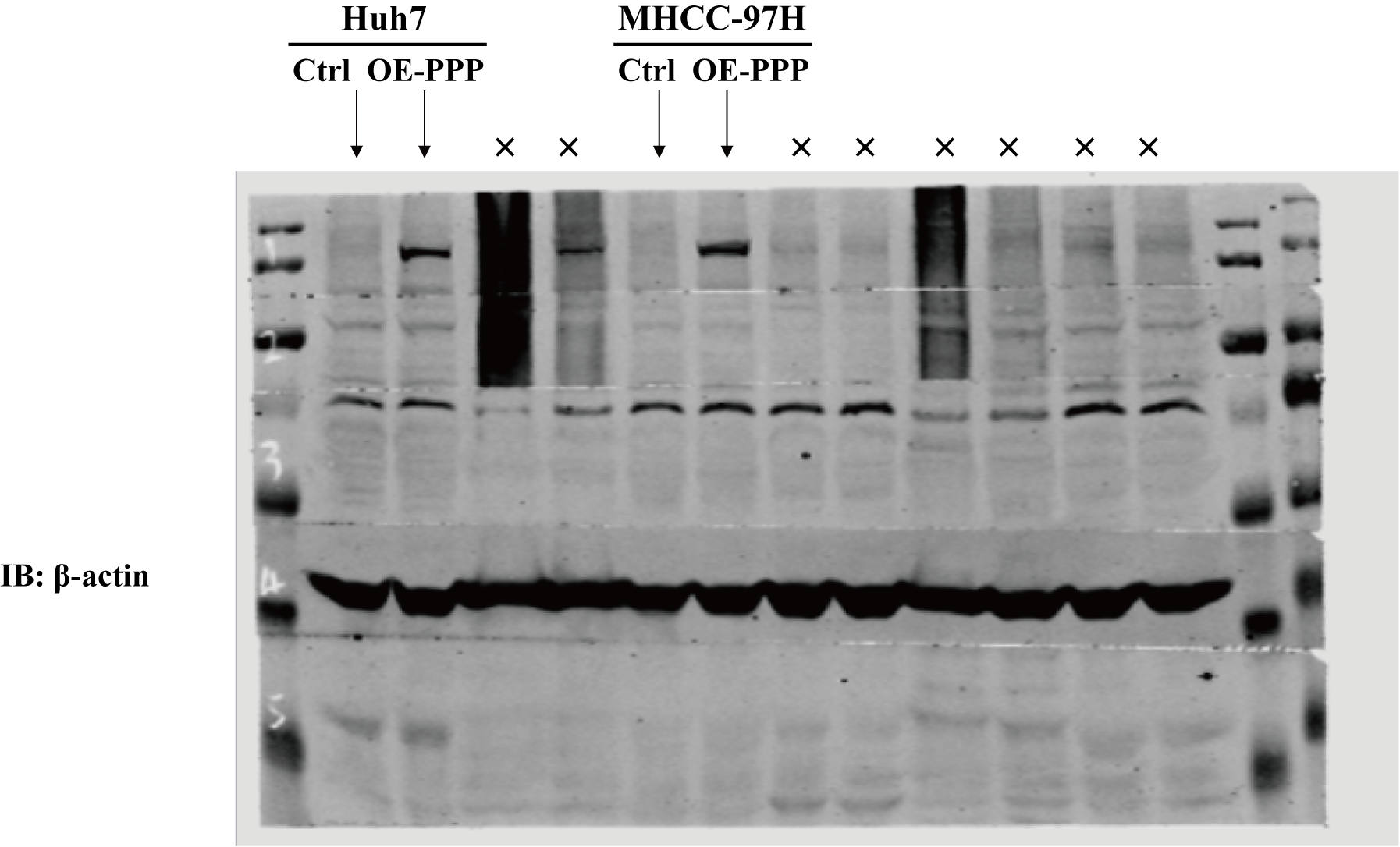

Supplement: Supplementary file 1 [file DataSheet1.zip › Supplementary file 3/WB images new/FigS1B-β-actin(PAK1).tif]

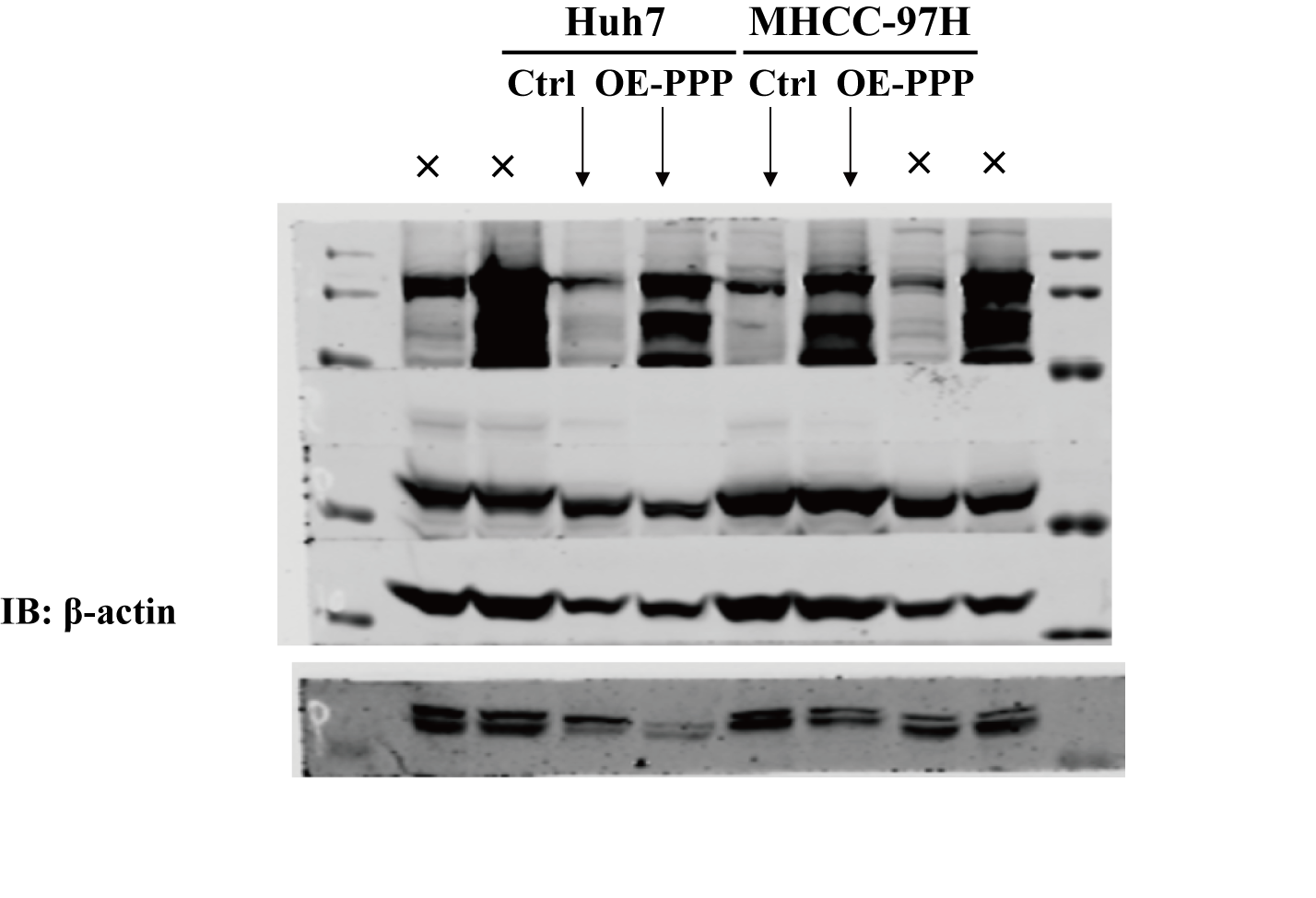

Supplement: Supplementary file 1 [file DataSheet1.zip › Supplementary file 3/WB images new/FigS1B-β-actin(PAK2).tif]

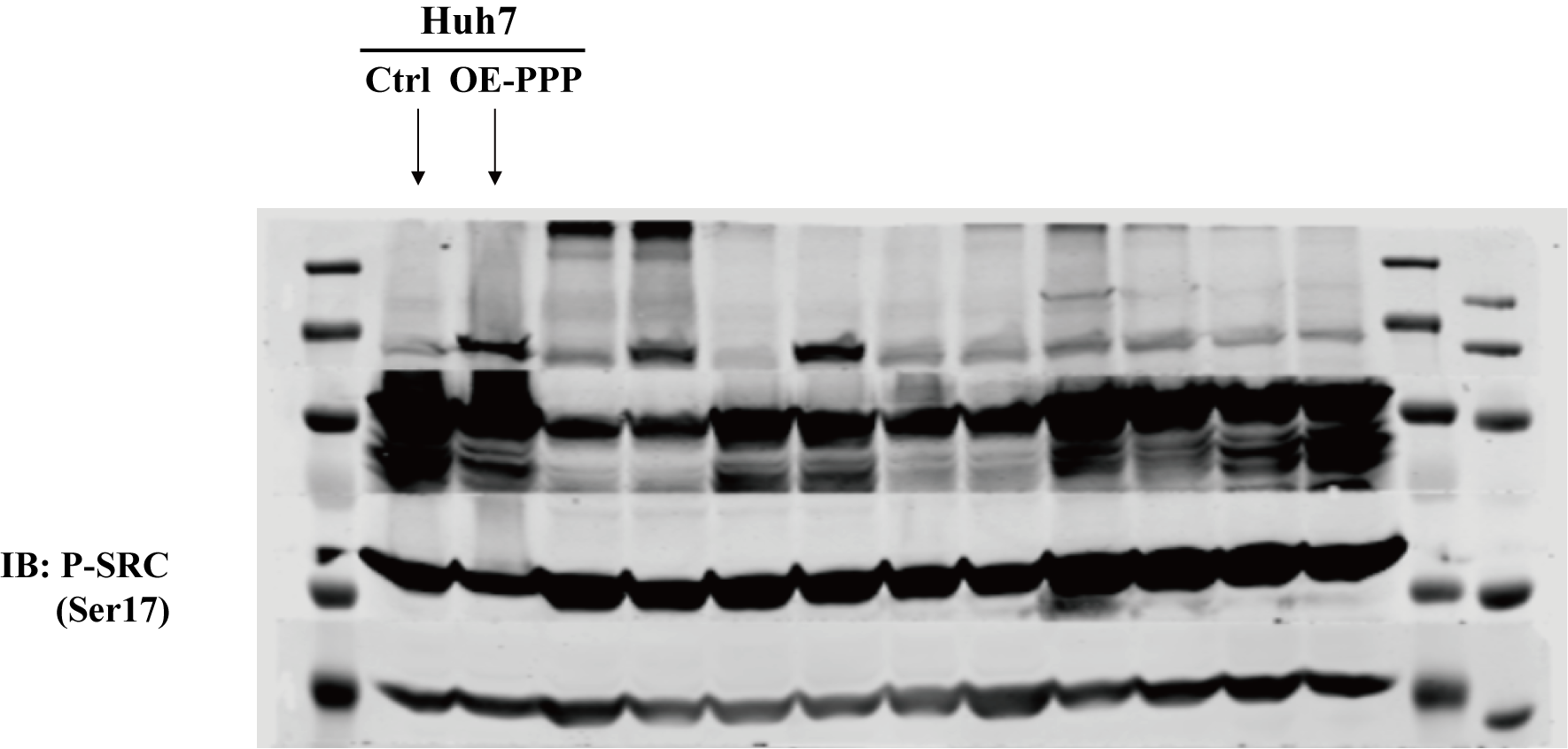

Supplement: Supplementary file 1 [file DataSheet1.zip › Supplementary file 3/WB images new/FigS1D-P-SRC.tif]

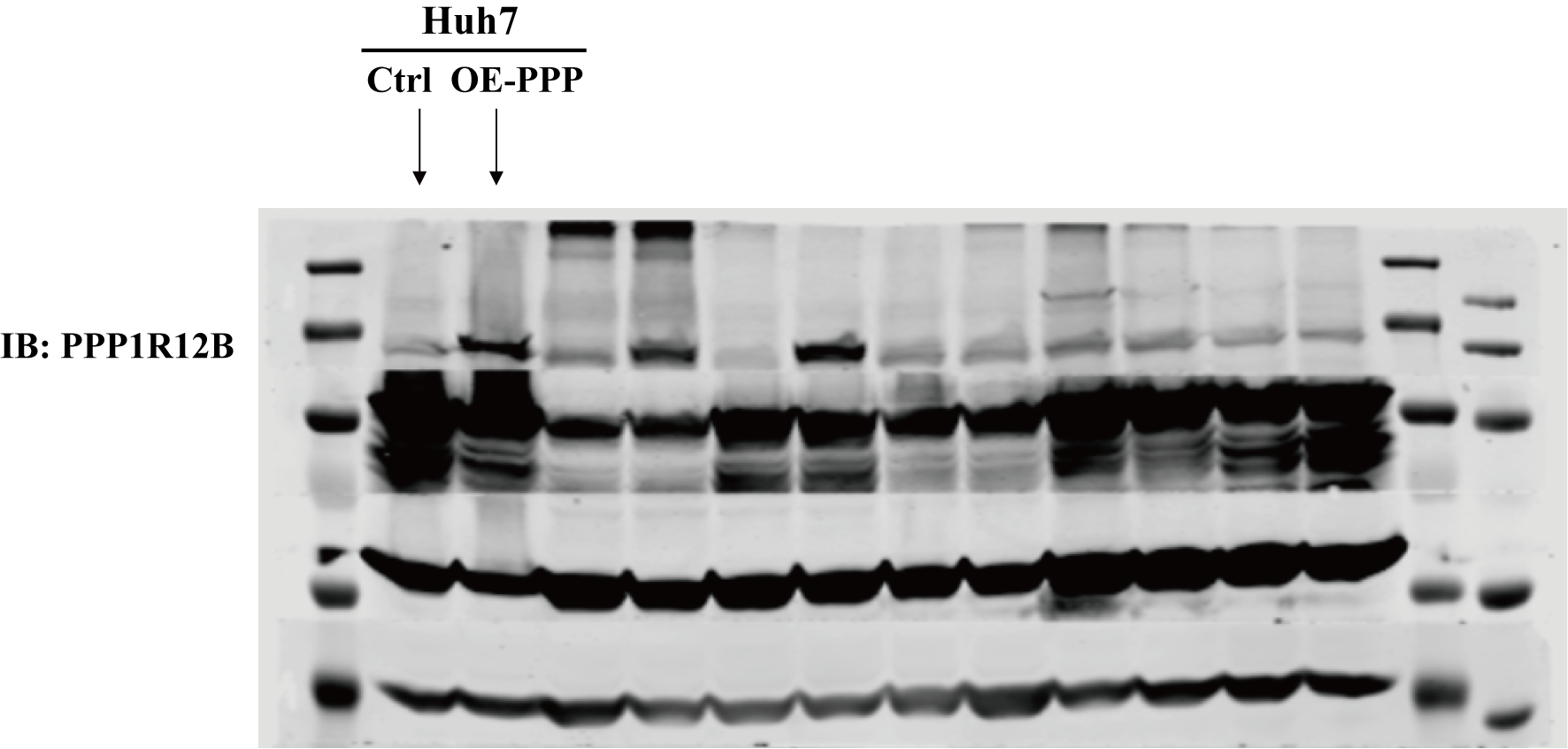

Supplement: Supplementary file 1 [file DataSheet1.zip › Supplementary file 3/WB images new/FigS1D-PPP1R12B.tif]

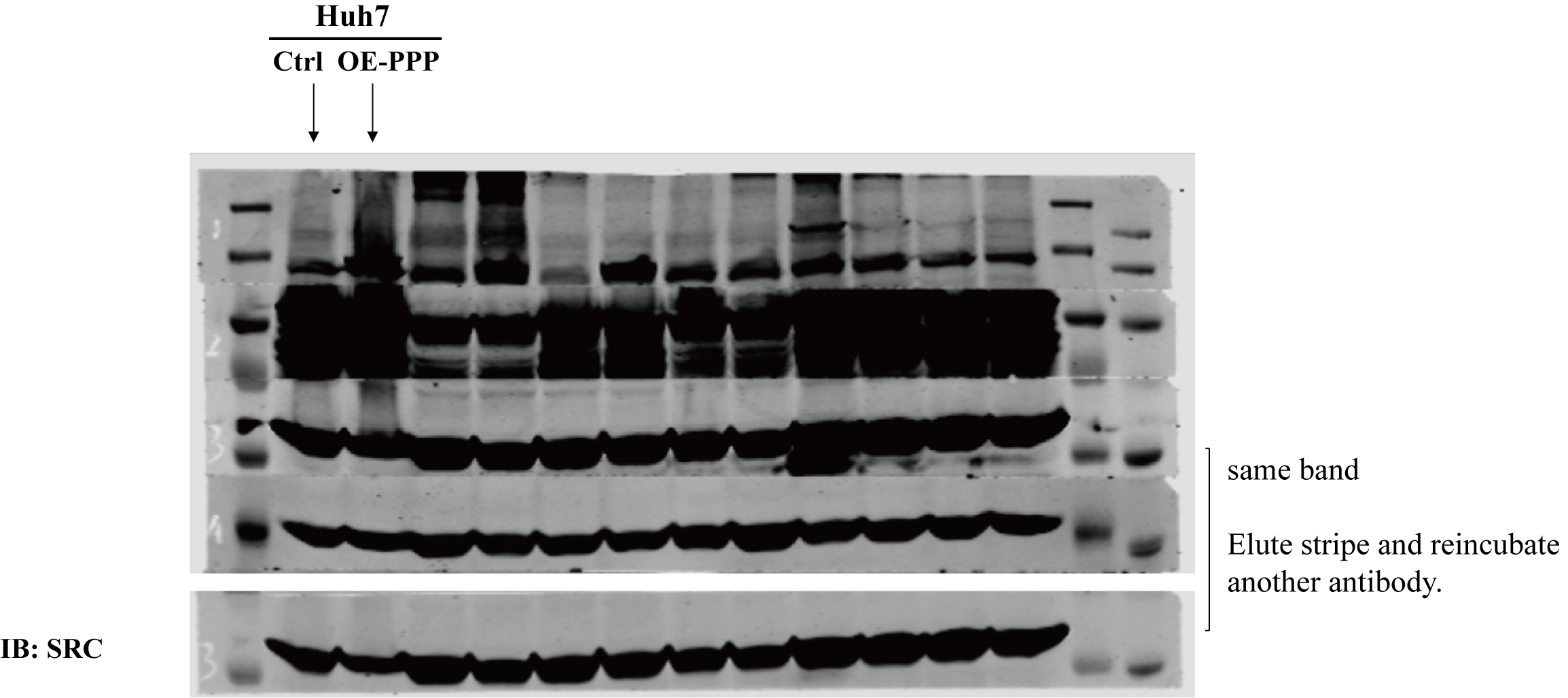

Supplement: Supplementary file 1 [file DataSheet1.zip › Supplementary file 3/WB images new/FigS1D-SRC-new.tif]

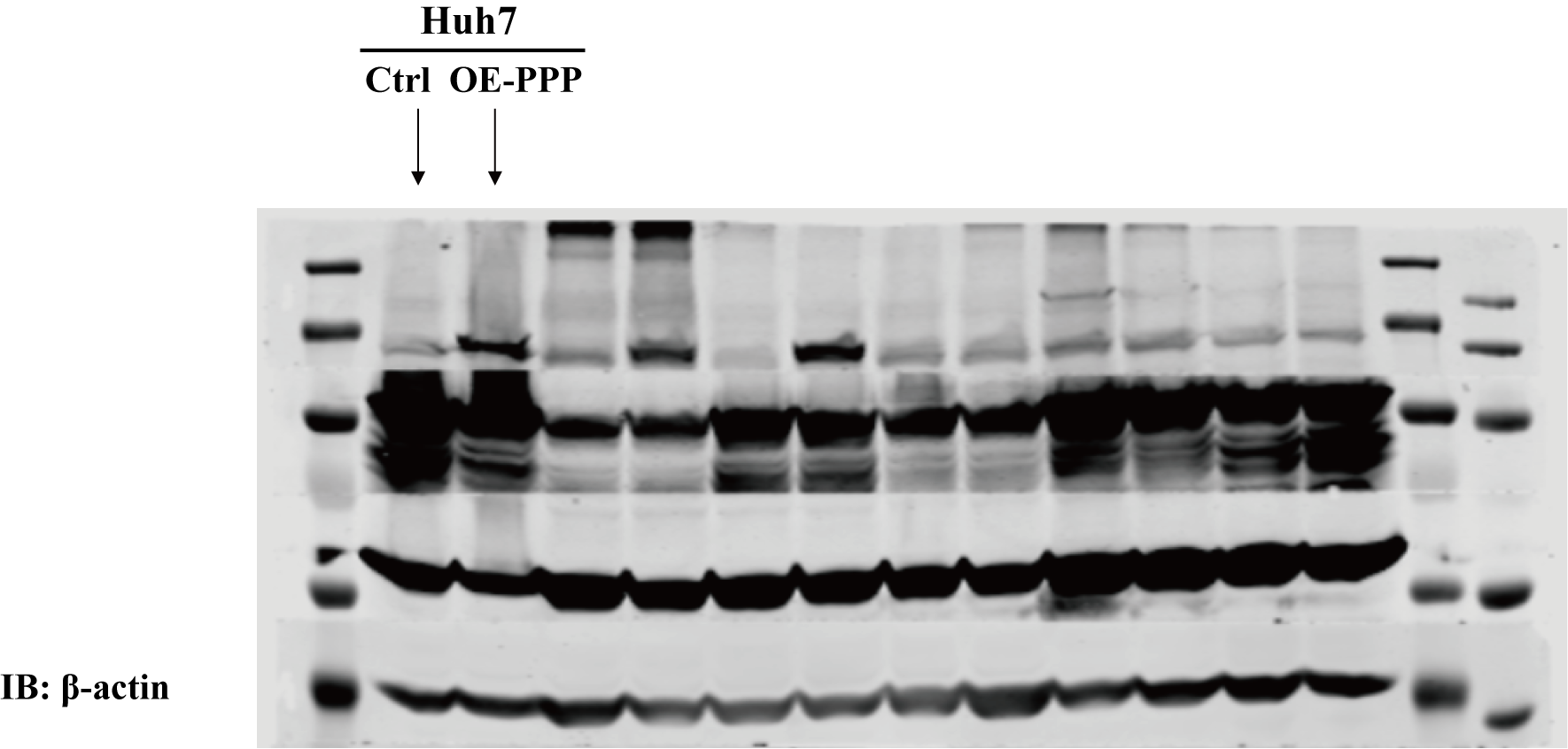

Supplement: Supplementary file 1 [file DataSheet1.zip › Supplementary file 3/WB images new/FigS1D-β-actin.tif]

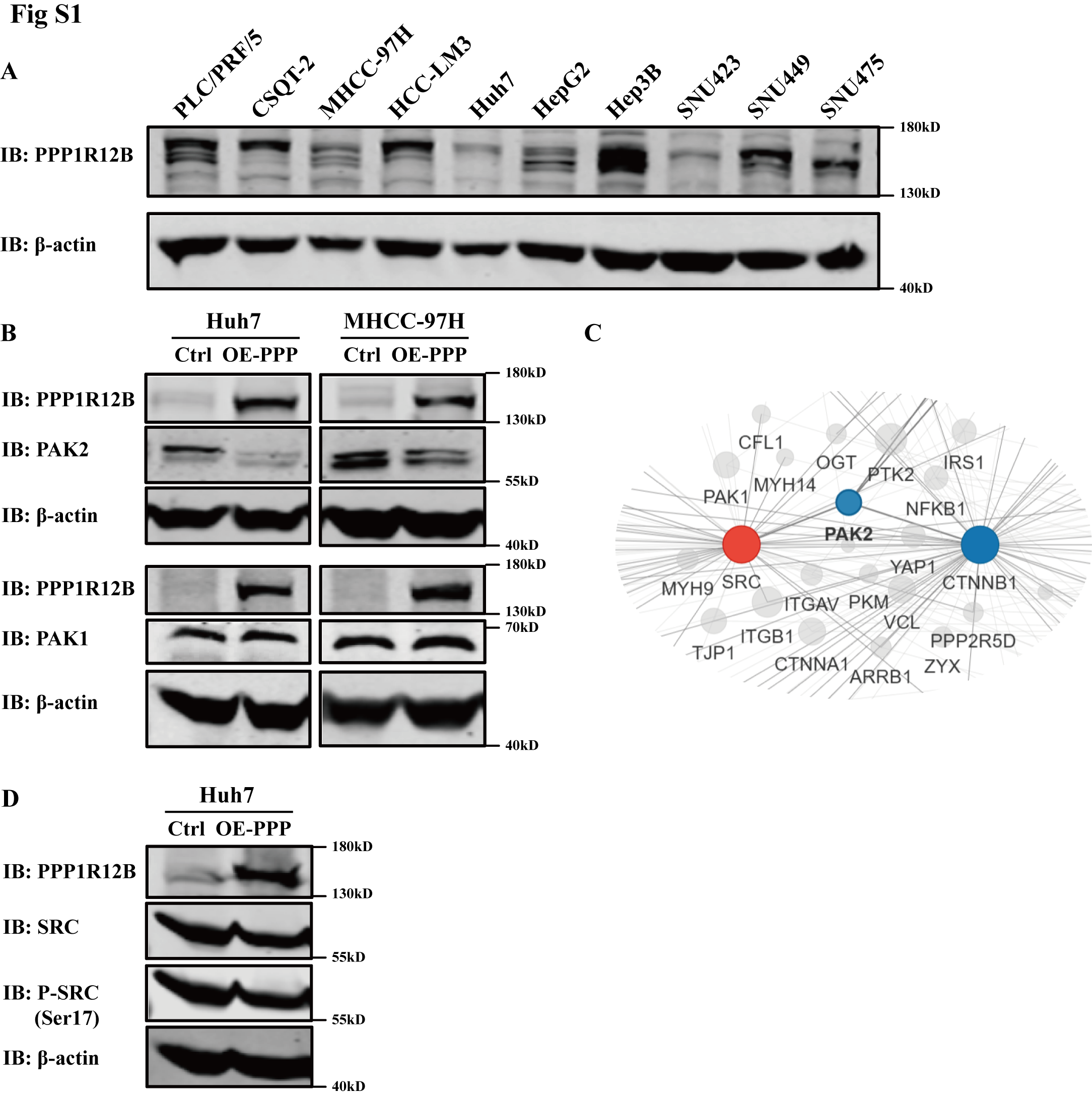

Supplement: Supplementary file 2 [file Image1.tif]
